# Drowning our sorrows: clinical and ethical considerations of termination in alcohol-affected pregnancy

**DOI:** 10.1186/s12884-020-03012-9

**Published:** 2020-06-10

**Authors:** Roger Martin, George Bruxner, Gary Ng, Catherine Brewster, Alka Kothari

**Affiliations:** 1 Redcliffe Hospital, Anzac Avenue, Redcliffe, Queensland 4020 Australia; 2 Department of Obstetrics & Gynaecology, Redcliffe Hospital, Anzac Avenue, Redcliffe, Queensland 4020 Australia; 3 Metro North Mental Health Service, Herston, 4029 Queensland Australia; 4 Faculty of Medicine, The University of Queensland, St Lucia, 4067 Queensland Australia; 5 Department of Alcohol & Other Drugs, Redcliffe Hospital, Redcliffe, 4020 Queensland Australia

**Keywords:** Pregnancy, Alcohol drinking, Termination of pregnancy, Medical ethics, Health behaviour, Women’s health

## Background

Clinicians caring for pregnant women who consume alcohol face many ethical considerations. They are obligated to provide non-judgmental and non-directive advice. Although current practice is guided by existing clinical guidelines [1–5] these are based on limited scientific evidence [6]. There is no objective and readily available test to corroborate subjective patient reports of alcohol use to predict the risk of alcohol-related adverse foetal outcomes. Additionally, the effects of alcohol exposure on the developing fetus are not completely understood [7].

## Main text

We present two case studies drawn from the authors’ place of practice, a 270-bed public outer metropolitan hospital serving a socio-economically disadvantaged region in Queensland, Australia [8]. There is a relatively higher rate of utilisation of hospital services by the residents in this growth corridor with a population of approximately 151,000 residents [9].

The two cases were selected as they illustrated two conflicting scenarios raising moral and ethical dilemmas for the treating teams. Consent was obtained from both patients for use of deidentified clinical information. The case studies were deemed exempt from full ethical review by The Human Research Ethics Committee of The Prince Charles Hospital in Brisbane, Australia (Project ID 51897, supplement 1).

### Case 1

Case one was a 26-year-old woman with two children born via caesarean section: a daughter born prematurely at 36 weeks and a son at 39 weeks, both for intrauterine growth restriction. Her son had a history of developmental problems and had been diagnosed with autistic spectrum disorder. She began drinking alcohol at age 13, described herself as an alcoholic and reported a strong family history of alcohol abuse. She participated in binge drinking and smoking tobacco and cannabis before and during her pregnancies. She was living alone with her two children following a relationship breakdown with the father of her son. There was uncertainty regarding the paternity of her current pregnancy, which was unplanned.

At 22 weeks of her gestation, she reported a typical alcohol intake of 10 to 40 standard drinks per day along with cannabis use and cigarette smoking. She felt that alcohol use during her previous pregnancies had not affected the health of her two children, nor had she been personally affected by her own mother’s drinking during the pregnancy. A live baby boy weighing 2620 g (growth restricted) was delivered by elective caesarean section at 38 weeks gestation.

### Case 2

Case two was a 35-year-old primigravida woman referred for early obstetric antenatal care, due to a high risk of Trisomy 21 based on the combined first trimester screen. There was a background of perceived infertility in the setting of endometriosis and irregular periods. Additionally, there was a history of deliberate self-harm, anxiety, obsessive-compulsive disorder and depression. She was currently prescribed medication for mental illness. She was in a stable relationship with a supportive partner, although the partner also had problems with alcohol dependence.

During the first few weeks of her pregnancy, she reported an alcohol intake of up to 20 standard drinks per day. This reduced to approximately 4 standard drinks per day post pregnancy awareness. Amniocentesis was performed for a high risk first trimester screen, and no aneuploidy was diagnosed. The patient requested a termination of pregnancy on the grounds of a perceived increased risk of foetal alcohol spectrum disorder (FASD). Following tertiary Maternal-Foetal Medicine (MFM) unit counselling and psychiatric assessment, a termination of pregnancy was performed to prevent serious danger to the woman’s mental health, thereby fulfilling the circumstances prevailing at the time to lawfully perform a termination in the state of Queensland, Australia [10]. She underwent a medical termination of pregnancy at 17 weeks gestation, although six months later, feelings of ongoing grief compunded by guilt and loss had led to a further increase in alcohol abuse.

## Method

A thorough search of the relevant databases including Medline, Embase and Pubmed was performed to evaluate the published literature on alcohol use and pregnancy. A wide-ranging consideration of the published epidemiological and observational evidence and existing guidelines is discussed and is complemented by clinical and ethical considerations.

## Conclusions

### There is no recognised safe level for alcohol consumption during pregnancy

Some studies have demonstrated a dose-response relationship between alcohol use in pregnancy and the spectrum of adverse pregnancy outcomes including FASD [11]. Although research is conflicting for some outcomes with modest alcohol use, e.g. for increased risk of preterm birth, [12] it appears that even low levels of alcohol consumption can lead to negative consequences, including spontaneous abortion, small for gestational age fetus and a low Apgar score [11, 13–15]. The relationship with small for gestational age was a persisting feature of case one. Complicating the situation further is the likelihood of under reporting of rates of alcohol consumption which was evidenced by a recent study estimating alcohol consumption from maternal hair samples [16]. Although, maternal alcoholism has been associated with an increased risk of miscarriage, this relationship is complex. A miscarriage can have detrimental effects on mental health, which may in turn precipitate excessive drinking [17]. This was somewhat illustrated in the second case study.

The methods for detecting foetal morbidity prior to delivery are limited with insufficient specificity of changes in cranio-facial and brain morphology observable in antenatal ultrasonography to allow for prenatal diagnosis [18]. This leads to few opportunities to objectively identify a foetus at risk of developing FASD to offer appropriate and informed advice.

The relationship between alcohol exposure and adverse outcomes is further weakened by research demonstrating a significant variation in alcohol consumption both within and between diagnostic groupings of mothers bearing children later diagnosed within the FASD continuum [19]. Adding to this confusion, there appear to be different consequences for regular low alcohol use compared with binge-drinking in pregnancy and for different types of alcoholic beverages [20].

Some authors suggest, as a consequence of the likely small margin between hazardous and non-hazardous drinking, that it is morally and ethically unacceptable for policies and guidelines to condone any consumption of alcohol during pregnancy [21]. Other authors feel that supporting a blanket ban on alcohol use in pregnancy is a hasty response given the lack of strong evidence with low levels of alcohol intake [22]. A recent meta-analysis described a paucity of research into the effects of light drinking, and a subsequent lack of confidence in published recommendations [12].

### Alcohol use when pregnant is related to their alcohol use when not pregnant

Prior published research has suggested that most women (85%) modify their drinking habits on recognition of pregnancy [23]. However, more recent research, has indicated that most women (82%) continued to drink through their pregnancy, [24] and that almost half (46%) continued risky drinking in their pregnancy [20]. The Australian guidelines changed in 2009 to promote complete abstinence from alcohol. Unfortunately, most women (72%) do not comply with these guidelines and pre-pregnancy alcohol behaviour remains the strongest predictor of alcohol avoidance in pregnancy [25]. The conclusion that pre-pregnancy alcohol consumption is associated with drinking during pregnancy was supported by a systematic review in 2011 [26].

### Women in vulnerable groups are most at risk of alcohol abuse during pregnancy

Women least likely to reduce their alcohol use during pregnancy were those with vulnerability factors including being younger, a heavy smoker, financially stressed, and having lower levels of education, poor mental health, low social support and exposure to domestic violence [27, 28]. A history of abuse or exposure to violence is consistently predictive of alcohol use in pregnancy [26].

Women in vulnerable groups may have reduced uptake of contraception and higher rates of unintended pregnancies. Australian research looking at women enrolled in opioid treatment programs found only 55% of sexually active women who were not planning a pregnancy were using contraception [29]. Within this population, the rate of unintended first pregnancy varied from 57 to 91%, with women who were younger in their first pregnancy being more likely to unintentionally fall pregnant [29]. This compares with Australian research on the general population, that has found that 40% of women have had an unintended pregnancy [30].

Women with unintended pregnancies may react to their pregnancy differently, and this may also influence their alcohol consumption during pregnancy. From Canadian data, women who reported indifference or unhappiness toward their pregnancy were significantly more likely to drink as a response [31].

### Women’s attitudes and the advice that they receive regarding alcohol consumption, is varied

Pregnancy is a special period in life that is often associated with motivation for women to improve their health in order to deliver a healthy child. This includes cessation of risky behaviours with negative health outcomes such as smoking and drinking. Women are significantly influenced by clinician advice during pregnancy, [32, 33] and as such, pregnancy offers clinicians unique opportunities to encourage their patients to address unhealthy behaviour [34, 35].

Research has however demonstrated that women are uncertain about the risk of alcohol consumption in pregnancy and perceive that the recommendations lack an evidence-base. In addition they feel pressure from friends, family and health professionals to both drink and not drink during their pregnancies [32, 36]. For most women, there is a delay between conception, pregnancy recognition and provision of information by a clinician regarding alcohol consumption consequently increasing the risk of inadvertent alcohol exposure [37].

Clinician advice is varied, both in the amount and content, and is perhaps proportionate to the clinicians’ own perception of risk and influenced by their own personal alcohol use and values about alcohol [38–40].

Women who decide to drink alcohol during pregnancy often discount abstinence messages and report a process of internal bargaining on issues such as the stage of their pregnancy and the type of beverages they consumed [36].

Realistically, achieving compliance with total abstinence for many patients may prove to be too difficult, leading clinicians to strive for minimising the level of alcohol consumption instead. While this may seem to be a pragmatic compromise in practice, there is no strong evidence to show that successfully achieving low alcohol consumption is a better outcome than failure at alcohol abstinence.

Promoting total abstinence implies a level of certainty that does not exist and carries perceived or true paternalism. Additionally, asserting total abstinence may increase under-reporting of alcohol use and fear associated with alcohol use in pregnancy, the latter resulting in “knee-jerk” reactions including termination of pregnancy [22].

### Clinical challenges for the obstetrician

Clinicians face personal, ethical and legal pressures when offering or refusing termination procedures to patients, including willingness to perform procedures, indications for termination and a gestational cut-off.

It is governed by both legal and ethical standards, creating time pressure to consider, offer and perform a termination. In Australia, the legality of termination is governed by state-based laws with considerable interstate variability. There is a dearth of research into how women seeking a termination access this service. Some published reports have suggested that women requesting a termination on the grounds of alcohol use have unintended pregnancies for other reasons as well [41–43].

#### Ethical issues related to the case studies

The three core elements of professional ethics that could be considered include:

- the acquisition and application of knowledge and professional skills,
- the provision of expert care to those in need, and
- the professional attributes of trustworthiness and efficacy enhancing the common good [44].

Ethical models take varying perspectives. Table 1 shows ethical tenets especially pertinent to the two cases included in this paper.

**Table 1:** Ethical tenets relating to case discussions

**Deontological ethics**	Rules based ethics, the ethics of the action itself, not the consequences of the action-individual focus	e.g. autonomy is absolute	
**Utilitarian/Teleological/Consequential ethics**	The consequences of actions take precedence-societal focus	e.g. autonomy may need to be curbed for the collective good	Includes: ***Virtue Ethics***-based on considered judgement, wisdom to achieve the “golden mean” ***Care Ethics***- ethical decisions are always made in a specific interpersonal context

The clinical circumstances of case one are associated with considerable clinical frustration. The clinician’s inability to utilise their knowledge and skill to intervene in a situation where a vulnerable woman and her unborn child are clearly at risk, engenders professional and clinical tensions. While the ethical principles of beneficence and non-maleficence should always be foremost in considerations of care circumstances (such as those illustrated by case one), they also create ambiguity with the conflict between the need to affirmatively meet the mother’s need (for a termination) versus the risk of ongoing harm (to the unborn child). The treating clinician also needs to juggle conflicting ethical tenets including the mother’s autonomy to decide to bear a child despite her lifestyle choices, against the effects of her alcohol dependence on her capacity to reason and fully appreciate the risks to her and her unborn child. This is further complicated by limitations in the knowledge base regarding risk and the lack of a definitive screening test. Medical professional ethics involve a social contract, [44] and the birth of a child with FASD is likely to have significant implications for precious societal resources as well as personal resources for the mother and her family.

Despite all these considerations the reality for the doctor is that there are likely to be very few avenues for action. Unlike termination of pregnancy, continuation of pregnancy, even an extremely hazardous one, is unlikely to be contested except perhaps in the most severe case of maternal incapacity, for example severe maternal intellectual disability. Statutory obligations, specifically to notify authorities of a high potential of risk to an unborn child, may incidentally meet the social contract to some extent, but ultimately the circumstances restrict the medical professionals’ influence on events.

There are a small number of jurisdictions in the USA that can impose severe restrictions on women with substance use disorders to prevent them drinking and a recent review of legislation related to maternal drinking in that country evidenced increasingly punitive approaches to pregnant women consuming alcohol [45, 46].

The least unsatisfactory, least damaging and most realistic option is for a pregnancy to proceed and for authorities to become involved if there is on-going risk, especially to the new born child. In case one, the ethical duty of the medical practitioner was to ensure appropriate postnatal care and protection measures were put in place. On the other hand, in case two, .....Please proceed with the below paragarph below

This paragraph the contemporary Queensland law supported the decision to terminate the pregnancy (termination was allowed where there is serious risk to the mother’s physical or mental health). Clearly the clinician in this situation had a professional and ethical responsibility to fully inform the patient of the current state of knowledge of the risks of alcohol use in pregnancy. In addition, it was imperative to assess her for possible anxiety or depressive conditions that may influence decision-making and look at remediable social issues potentially contributing to a negative perception of the pregnancy. However, with the current state of knowledge, it would not be ethical to provide full reassurance or to thwart a competent patient’s autonomy to proceed with a legally justified termination.

The issues therefore relate to the clinicians’ own struggle with the decision to terminate the pregnancy, especially due to the possibility that a potentially normal foetus may to be aborted. An author of this paper described the situation as playing on their personal and professional moral code. In many jurisdictions, it is acceptable for clinicians to defer responsibility to colleagues if they are unable to justify performing a termination on the grounds stated. In our case, the clinician agreed to proceed with the termination, but this did not come without significant clinical angst. The support of MFM and psychiatry with case two helped to establish an unbiased and collective opinion that satisfied the legal workaround but perhaps did little to share the ethical burden.

Virtue ethics [44] may offer clinicians an ethical indicator in considering their struggles with their patients’ wishes and their consequences. Clinicians takes a reflective stance considering their own experiences and possible biases influencing their view, and adopt an empathic stance, putting themselves in their patient’s position in trying to understand their wishes and circumstances. The related concept of care ethics [44], based on the emotional bond with the patient, may be relevant after the decision has been made, to help the woman through the process.

Alcohol use during pregnancy appears to be related to alcohol use prior to pregnancy. Providing care for pregnant women indulging in significant alcohol consumption and performing a termination of pregnancy is challenging and presents many ethical dilemmas. There is neither a recognised safe level of alcohol consumption in pregnancy, nor an objective method to corroborate self-reported rates of alcohol consumption. Recent guidelines, such as those of the United Kingdom, [6] recommend abstinence as the safest option, but historically the evidence suggests most women do not comply with complete abstinence. Women in vulnerable groups are most at risk of alcohol abuse during pregnancy. There is also uncertainty about alcohol recommendations in pregnancy due to varied advice from clinicians. A delay between conception and recognition of pregnancy can further increase the risk of inadvertent alcohol exposure. Practitioners should offer best supportive and qualitative evidence, provide non-judgmental, non-directional advice, and promote alcohol reduction and social support. We hope that these cases help highlight the ethical and moral dilemmas faced by health practitioners and lead to greater support to better share the ethical burden.

## Data Availability

Not applicable.

## Abbreviations

FASD: Fetal Alcohol Spectrum Disorder
MFM: Maternal Foetal Medicine
